# The mediating and moderating effects of resilience between childhood trauma and geriatric depressive symptoms among Chinese community-dwelling older adults

**DOI:** 10.3389/fpubh.2023.1137600

**Published:** 2023-04-14

**Authors:** Shaojie Li, Yongtian Yin, Guanghui Cui, Chi Zhang, He Zhu, Yao Yao

**Affiliations:** ^1^School of Public Health, Peking University, Beijing, China; ^2^China Center for Health Development Studies, Peking University, Beijing, China; ^3^Shandong University of Traditional Chinese Medicine, Jinan, China; ^4^Department of Integrated Traditional Chinese and Western Medicine, Peking University First Hospital, Beijing, China; ^5^The Key Laboratory of Geriatrics, Beijing Institute of Geriatrics, Institute of Geriatric Medicine, Chinese Academy of Medical Sciences, Beijing Hospital, National Center of Gerontology of National Health Commission, Beijing, China

**Keywords:** childhood traumatic events, childhood trauma severity, resilience, depressive symptoms, older adults, mediation, moderation

## Abstract

**Objective:**

This study aims to examine the association between childhood traumatic events (CTEs), childhood trauma severity, and depressive symptoms, as well as to examine the mediating and moderating roles of resilience in these associations.

**Methods:**

We conducted a cross-sectional study of 1,091 community-dwelling older adults in Jinan, China. The trauma history questionnaire (THQ) was used to measure CTEs and childhood trauma severity. CTEs were defined as the number of traumatic events before the age of 18. We calculated childhood trauma severity by multiplying the number of CTEs by the participants’ self-perceived impact level of the events from the THQ. We then applied the 15-item Geriatric Depression Scale and 10-item Connor–Davidson Resilience Scale to assess participants’ depressive symptoms and resilience, respectively. Linear regression models were used to examine the associations, and structural equation modeling was used to examine the mediating and moderating roles of resilience.

**Results:**

Childhood traumatic events, childhood trauma severity, and resilience were all associated with depressive symptoms in older adults. Resilience mediated the relationship between childhood trauma severity and depressive symptoms (*β* = 0.082, 95% CI = 0.045–0.123), accounting for 26.6% of the overall effect (*β* = 0.308, 95% CI = 0.190–0.422). However, there was no evidence that resilience mediated the association between CTEs and depressive symptoms. In addition, we did not find that resilience played a moderating role in the associations of CTEs, childhood trauma severity with depressive symptoms.

**Conclusion:**

Resilience plays a mediating role in the relationship between childhood trauma severity and depressive symptoms. Intervention measures on improving resilience may reduce childhood trauma severity associated with depression risk in older Chinese adults.

## 1. Introduction

Depression, the most common mental illness worldwide (1), is one of the leading causes of disability and death (2). The prevention and control of depression are critical global public health challenges. Older adults are especially susceptible to depression; a systematic review showed that the global prevalence of depressive symptoms among older adults was 31.74% (3). The increasing aging of China’s population has heightened scholars’ and governments’ concerns about depression in older adults. Several systematic reviews have shown that the prevalence of depressive symptoms among older Chinese adults exceeds 20% (4–6). Depression not only deteriorates the health of older adults but can also pose a substantial mental and economic burden on them and their families (7). Given the high prevalence and adverse effects of depression, exploring its risk factors is of great significance for its future prevention and control.

Emerging research in recent decades has focused on the risk factors for depression from the life course and cumulative risk perspectives (8). According to these perspectives, early life events cumulatively affect individuals throughout their lifespans (9, 10). At present, multiple studies have explored the association between adverse childhood experiences (ACEs) and depression in adulthood and old age. Childhood trauma, one of the main types of ACEs, has received considerable attention from scholars in recent years (11). Childhood trauma refers to physical or psychological damage to individuals before the age of 18 caused by external factors, including abuse, neglect, exposure to violent places, and any type of exploitation (12–14). A nationwide epidemiological study in the US showed that older adults with childhood physical or psychological abuse tend to suffer from psychiatric disorders, including depression (15). An Irish study also determined that ACEs (e.g., abuse, neglect, and household dysfunction) were associated with a higher risk of late-life depressive symptoms (16). Other researchers have also observed the same association in Asian countries. Research in Japan has shown that ACEs are associated with the onset of depressive symptoms in adults aged ≥65 (17). A study based on the china health and retirement longitudinal survey (CHARLS) suggested that middle-aged and older adults who experienced parental physical abuse or emotional neglect in childhood were at a higher risk of depressive symptoms (18). However, it should be noted that not all individuals with ACEs develop depressive symptoms. Another CHARLS study did not find a statistically significant association between childhood neglect and abuse, and depressive symptoms in middle-aged and older adults (19). Given these inconsistent findings, it is necessary to further analyze the relationship between childhood traumatic events (CTEs) and depression in older adults to inform future prevention and intervention plans.

Previous studies have shown that individuals’ mental health is related not only to the occurrence of life events but also to their perceptions of the impact of those life events (20). Based on this idea, scholars have conceptualized the impact of CTEs as childhood trauma severity, which mainly reflects individuals’ subjective perceptions of the impact of CTEs on their lives (21, 22). The more individuals perceive that CTEs significantly affect their present lives, the more serious is the childhood trauma. Scholars are increasingly interested in the relationship between childhood trauma severity and poor mental health. For instance, a study of adolescents in China found that childhood trauma severity was associated with depressive symptoms (23). Similarly, a recent US study found that childhood trauma severity was significantly related to depressive symptoms in middle-aged people (24). However, it is still unclear whether this association applies to older adults. Therefore, further research investigating the association between childhood trauma severity and depressive symptoms in older adults is required.

Although we cannot return to childhood to change traumatic events, we can learn more about the mechanism of the association between childhood trauma and depressive symptoms in older adults. This understanding would provide a scientific basis for developing interventions to prevent or mitigate depressive symptoms in older adults with childhood trauma. Several studies have explored the possible mechanism of the association between childhood trauma and depressive symptoms in older adults. For example, a CHARLS-based study found that childhood neglect and abuse indirectly affected depressive symptoms in middle-aged and older adults through poor health and low socioeconomic status (19). The English Longitudinal Study of Aging found that plasma concentrations of C-reactive protein (CRP), an inflammatory biomarker, played a mediating effect in the association between ACEs and geriatric depression (25), that is, ACEs may lead to a rise in plasma CRP levels and subsequently increase depressive symptoms in older adults. However, these mediating roles identified in the above research were difficult to change in a short period of time, limiting the feasibility of designing interventions. Therefore, further exploration of the mechanism underlying the association between childhood trauma and depressive symptoms in older adults is crucial.

Numerous studies on children and adolescents have suggested that resilience might play an important role in the association between childhood trauma and depressive symptoms (26–29). Resilience refers to the ability to cope with, adapt, or surmount adversity and traumatic events (30, 31), which mainly involves two levels, biological and psychological. Biological resilience focuses on the ability to recover quickly and completely following deviations from normal physiological states or damage due to stressors or adverse health events, including the speed and quality of DNA repair, the ability to restore glucose levels or blood pressure back to normal after deviation caused by a stressor, the ability to quickly heal a wound, etc. (32). Psychological resilience reflects an individual’s positive psychological resources and psychological traits in coping with and adapting to stress, involving self-efficacy, hope, emotional regulation, etc. (33, 34). At present, research on biological resilience is mainly conducted in the fields of geriatric medicine, disease treatment and rehabilitation (32), while research on psychology and behavior has focused on psychological resilience (35). In this study, we only focused on the psychological level of resilience and did not involve biological resilience. A recent multivariate meta-analysis showed that resilience mediated the association between childhood trauma and depression (36). However, this meta-analysis did not include the older adult population; thus, we cannot generalize its findings to older adults. To our knowledge, only one study has assessed the mediating role of resilience in the association between ACEs and geriatric depressive symptoms (37). However, that study’s findings suggested that resilience did not mediate the association (37). Therefore, it is still necessary to further explore whether resilience has a mediating effect in the above associations.

A recent systematic review that included 13 observational studies over nearly 30 years found that resilience not only mediates the association of childhood adversity with depressive symptoms, but also plays a moderating role (38). However, similar to a previous meta-analysis (36), this systematic review also did not incorporate findings of older people. The sensitization hypothesis of childhood trauma suggests that it might increase individuals’ sensitivity to adverse events, reduce their ability to adapt to stress, and increase the likelihood of developing psychopathology in the future (39). The multisystem development framework for resilience also posits that multiple systems and factors influence resilience, including individual characteristics, external environments, and early and current stressful life events (40). Meanwhile, the protective model of resilience states that resilience buffers the negative effects of stressful events and adversity, reducing an individual’s risk of adverse outcomes (41). Given these perspectives, we assumed that older adults who have experienced childhood trauma might have reduced resilience to trauma or stressful events, increasing the risk of psychological problems later in life. In addition, we also hypothesized that high resilience levels would reduce the association of childhood trauma with adverse mental health among older adults.

This study had two objectives: (1) to explore the association between childhood trauma (CTEs and childhood trauma severity) and depressive symptoms in older adults from a life course perspective and (2) to explore whether resilience mediates or moderates the association between childhood trauma (CTEs and childhood trauma severity) and depressive symptoms in older adults.

## 2. Materials and methods

### 2.1. Participants

We conducted a cross-sectional study in Jinan city, China, in December 2019. In 2020, the population of Jinan city was 9.20 million, of which 19.96% were aged 60 and above. We calculated the epidemiological sample size using this formula to estimate the proportion of binary outcomes:$n=\frac{Z_{\alpha /2}{}^{2}P\left(1-P\right)}{\delta^{2}}$(42). A previous meta-analysis showed that the prevalence of depressive symptoms among Chinese older adults was 23.6% (5). Therefore, we set the values as follows: *p* = 0.23.6, α = 0.05, *μ*_α/2_ = 1.96, and *δ* = 0.03, which required a minimum sample size of 770 participants.

Using a stratified cluster sampling method, we randomly selected three districts and one county from Jinan as the primary sampling areas. Second, we selected two streets or townships from each district and county as secondary sampling areas. Third, we randomly selected two communities or administrative villages from six streets or towns as the tertiary sampling areas. Finally, we surveyed all older adults in six communities and 10 administrative villages using the following inclusion criteria: age ≥ 60 years; live locally for at least six months; no hearing and language impairment; no major diseases; informed consent; and voluntary participation in the survey. The exclusion criteria were serious cognitive impairment and major depression (as reported by family members). We recruited 1,130 participants, but excluded 39 because they did not meet the criteria. Thus, 1,091 older adults completed all the surveys. This study conformed to the recognized standards under the Declaration of Helsinki. We obtained written informed consent from all participants prior to the investigation. The Medical Ethics Committee of the Second Affiliated Hospital of Shandong University of Traditional Chinese Medicine approved this study.

### 2.2. Measures

#### 2.2.1. Childhood trauma

We assessed childhood trauma using the 15-item Trauma History Questionnaire (THQ-15) (43, 44), asking whether the participants had experienced any of15 types of trauma before the age of 18 (0 = no to 1 = yes). The participants self-assessed their childhood trauma severity by rating the impact of these events on their current lives, using a five-point Likert scale for each event (0 = not at all to 4 = very hard). CTEs were generated by the number of 15 traumatic events, which ranged from 0 to 15. We calculated the childhood trauma severity by multiplying the sum of 15 CTEs by the participants’ self-perceived impact level for these events (0–60 points). The Chinese version of the THQ-15 has been employed in childhood trauma surveys in the Chinese population and has demonstrated good reliability and validity (45). In this study, the Cronbach’s alpha coefficient for the THQ-15 was 0.785.

#### 2.2.2. Depressive symptoms

We adopted the 15-item Geriatric Depression Scale (GDS-15) to assess depressive symptoms in older Chinese adults. This scale comprised 15 items (0 = no to 1 = yes). The items’ sum is the total score (0–15). The GDS-15 has been translated into Chinese and has demonstrated good psychometric properties (46). In this study, the Cronbach’s alpha coefficient for the GDS-15 was 0.815.

#### 2.2.3. Resilience

We assessed resilience using the 10-item Connor–Davidson Resilience Scale (CD-RISC-10, 47). The participants scored each item on a five-point Likert scale (0 = never to 4 = almost always). We summed the 10 items for the total scale score (0–40); higher scores indicated higher individual resilience. The Chinese version of the CD-RISC-10 has been validated in older adults and has good reliability and validity (48). In this study, the Cronbach’s alpha coefficient of the CD-RISC-10 was 0.961.

#### 2.2.4. Covariates

We controlled for sociodemographic characteristics associated with depressive symptoms in older Chinese adults (49). These included age, sex (male or female), residence (urban or rural), marital status (married or unmarried), monthly household income (<2,500, 2,500–5,000, or > 5,000 yuan), education level (primary school and below, junior high school, or high school and above), and chronic disease status. The participants self-reported their chronic disease status from a list of common chronic diseases, such as hypertension, diabetes, asthma, and arthritis. The participants were included in the chronic disease group if they had suffered from any chronic disease.

### 2.3. Statistical methods

We described continuous variables that conformed to a normal distribution using the mean (standard deviation or SD) and continuous variables that did not conform to a normal distribution using the median and quartiles (25th and 75th percentiles or *P*_25_ and *P*_75_, respectively). We described categorical variables by frequency and constituent ratios, and used Pearson correlation analysis to analyze the relationship between childhood trauma, resilience, and depressive symptoms. We used structural equation modeling with the robust maximum likelihood and bootstrap method (repeat sampling 5,000 times) to test the mediating and moderating effects of resilience between childhood trauma and depressive symptoms. A statistically significant mediating effect of resilience was indicated when the 95% bias-corrected confidence interval (CI) for the indirect effect of childhood trauma on depressive symptoms through resilience did not include zero. Similarly, the significant association of the interaction term between childhood trauma and resilience with depressive symptoms (95%CI did not include zero) suggested that resilience mediates the association between childhood trauma and depressive symptoms. Our mediation and moderation models all adjusted for age, sex, residence, marital status, monthly family income, educational level, and chronic disease status. All descriptive and correlation analyses were performed using STATA, Version 17.0 (Stata Corp, College Station, TX, USA). Mediation and moderation model testing was performed using AMOS, Version 25.0 (IBM Corp., Armonk, NY, USA).

## 3. Results

### 3.1. Descriptive statistics

The mean age of the 1,091 older adults was 70.43 years (SD = 6.58). Men and women accounted for 58.2 and 41.8% of the sample, respectively. More than two-thirds (73.1%) of the older adults lived in rural areas, 79.6% were married, and 66.8% had an education level of high school below. More than half (65.8%) had a monthly household income of 2,500–5,000 yuan, and 58.4% suffered from chronic diseases. The mean GDS-15 total score was 3.91 (SD = 3.37), and the prevalence of depressive symptoms was 16.5% (180/1091). The median scores of CTEs and childhood trauma severity were 2.00 and 1.00, respectively. The mean CD-RISC-10 total score was 35.61 (SD = 7.48), ranging from 10.00 to 50.00. The additional details are provided in Table 1.

**Table 1:** Characteristics of the study sample (*N* = 1,091).

Variables	
**GDS**	
Total score, mean (SD)	3.9 (3.4)
>8, *n* (%)	180 (16.5)
CTEs, medium (*P*_25_, *P*_75_)	2.0 (1.0, 4.0)
Childhood trauma severity, medium (*P*_25_, *P*_75_)	1.0 (0.0, 3.0)
CD-RISC-10	25.6 (7.5)
Age, yeas, Mean ± SD	72.0 ± 7.0
**Sex,** ***n*** **(%)**	
Male	635 (58.2)
Female	456 (41.8)
**Residence,** ***n*** **(%)**	
Urban residency	294 (26.9)
Rural residency	797 (73.1)
**Marital status,** ***n*** **(%)**	
Unmarried	223 (20.4)
Married	868 (79.6)
**Educational level,** ***n*** **(%)**	
Uneducated	225 (20.6)
High school below	729 (66.8)
High school and above	137 (12.6)
**Monthly household income (RMB),** ***n*** **(%)**	
<2,500	261 (23.9)
2,500–5,000	718 (65.8)
>5,000	112 (10.3)
**Chronic disease,** ***n*** **(%)**	
Yes	637 (58.4)
No	454 (41.6)

### 3.2. Correlation analysis

Table 2 shows the results of the relationships among CTEs, childhood trauma severity, resilience, and depressive symptoms. The Pearson correlation analysis showed that CTEs (*r* = 0.145, *p* < 0.001) and childhood trauma severity (*r* = 0.213, *p* < 0.001) were all positively associated with depressive symptoms. In addition, the associations of CTEs (*r* = −0.069, *p* = 0.023) and childhood trauma severity (*r* = −0.176, *p* < 0.001) with resilience were also significant. Moreover, resilience was negatively associated with depressive symptoms (*r* = −0.410, *p* < 0.001).

**Table 2:** Correlation analysis for childhood trauma, resilience, and geriatric depressive symptoms.

Variables	1	2	3	4
1. CTEs	–			
2. Childhood trauma severity	0.722^***^	–		
3. Resilience	−0.069^*^	−0.176^***^	–	
4. Depressive symptoms	0.145^***^	0.213^***^	−0.410^***^	–

^*^Indicates *P* < 0.05; ^***^indicates *p* < 0.001.

### 3.3. Mediation and moderation analysis

**Figure 1:**
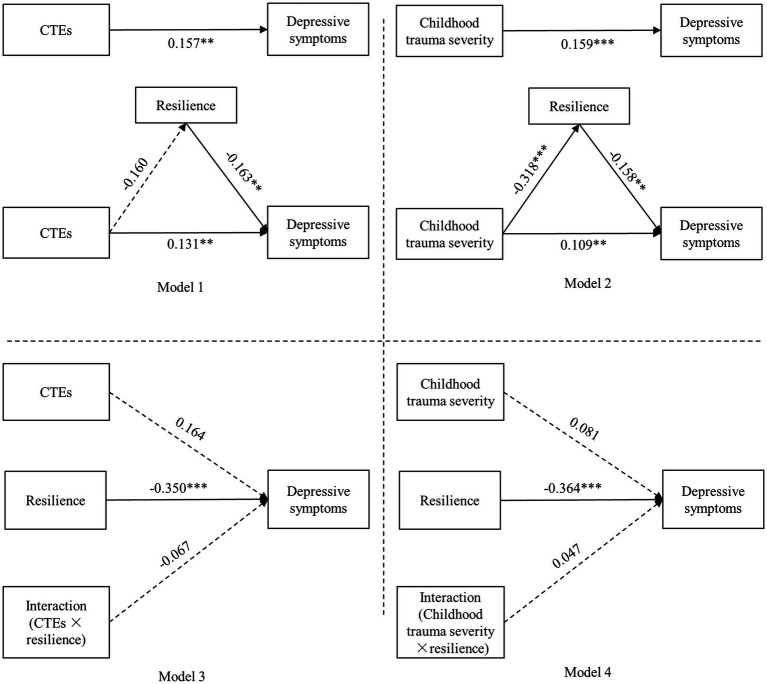
Test for the mediating and moderating effects of resilience in the association between childhood trauma and depressive symptoms. Model 1 and Model 3 were the mediation and moderation models of resilience in the associations of childhood traumatic events (CTEs) with depressive symptoms, respectively. Model 2 and Model 4 were the mediation and moderation models of resilience in the associations of childhood trauma severity with depressive symptoms, respectively. ^*^p < 0.05, ^**^p < 0.01, ^***^p < 0.001; all coefficients are unstandardized coefficients; the solid line represents the path that is statistically significant (*p* < 0.05), and the dashed line represents the path that is not statistically significant (*p* > 0.05).

We constructed four structural equation models to explore the mediating and moderating effects in associations of CTEs and childhood trauma severity with depressive symptoms. All models were adjusted for age, sex, residence, marital status, monthly family income, education level, and chronic disease status. In Model 1, there was no evidence that resilience mediated the association between CTEs and depressive symptoms (*β* = 0.026, 95% CI = −0.001–0.021, *p* = 0.075). In Model 2, the mediation analysis showed that resilience mediated the association between childhood trauma severity and depressive symptoms (*β* = 0.050, 95% CI = 0.027–0.077, *p* < 0.001), accounting for 31.5% of the total effect (*β* = 0.159, 95% CI = 0.093–0.225, *p* < 0.001). As for moderation analysis, the results of Model 3 and Model 4 showed the interaction CTEs and resilience (*β* = −0.067, 95% CI = −0.264–0.158, *p* = 0.554) and interaction childhood trauma severity and resilience (*β* = 0.047, 95% CI = −0.180–0.272, *p* = 0.625) were not significantly associated with depressive symptoms, indicating that the resilience could not moderate the associations of CTEs and childhood trauma severity with depressive symptoms. Further details are provided in Table 3 and Figure 1.

**Table 3:** Testing the mediating and moderating effects of resilience on the associations of childhood traumatic events (CTEs) and childhood trauma severity with geriatric depressive symptoms.

Model and path	*β*	SE	LLCI	ULCI	*P*
**Mediation model**					
CTEs→resilience→depressive symptoms					
CTEs→depressive symptoms	0.131	0.041	0.048	0.209	0.003
CTEs→resilience	−0.160	0.084	−0.322	0.010	0.064
Resilience→depressive symptoms	−0.163	0.014	−0.189	−0.136	0.001
**Effect**					
Total effect	0.157	0.043	0.070	0.239	0.001
Indirect effect *via* resilience	0.026	0.005	−0.001	0.021	0.075
Childhood trauma severity→resilience→depressive symptoms					
Childhood trauma severity→depressive symptoms	0.109	0.033	0.043	0.171	0.002
Childhood trauma severity→resilience	−0.318	0.075	−0.475	−0.178	<0.001
Resilience→depressive symptoms	−0.158	0.014	−0.184	−0.130	0.001
**Effect**					
Total effect	0.159	0.033	0.093	0.225	<0.001
Indirect effect *via* resilience	0.050	0.013	0.027	0.077	<0.001
**Moderation model**					
CTEs, resilience, and their interaction→depressive symptoms					
CTEs→depressive symptoms	0.164	0.104	−0.060	0.356	0.153
Resilience→depressive symptoms	−0.350	0.045	−0.431	−0.255	<0.001
Interaction CTEs and resilience→depressive symptoms	−0.067	0.108	−0.264	0.158	0.554
Childhood trauma severity, resilience, and their interaction→depressive symptoms					
Childhood trauma severity→depressive symptoms	0.081	0.121	−0.154	0.316	0.570
Resilience→depressive symptoms	−0.364	0.034	−0.428	−0.300	<0.001
Interaction childhood trauma severity and resilience→depressive symptoms	0.047	0.118	−0.180	0.272	0.625

SE, standard error; LLCI, lower limit confidence interval 95%; ULCI, upper limit confidence interval 95%.

## 4. Discussion

This study found positive associations of CTEs and childhood trauma severity with geriatric depressive symptoms. To our knowledge, this study is the first to explore the association between childhood trauma severity and geriatric depressive symptoms. In addition, we found that resilience mediated the association between childhood trauma severity and geriatric depressive symptoms, deepening our understanding of the association between childhood trauma and geriatric depressive symptoms and its mechanism, thus providing a scientific basis for future intervention studies.

We found a significant association between CTEs and depressive symptoms in older adults. The greater the number of CTEs, the more severe the depressive symptoms in older adults. This finding is consistent with previous studies (17, 18, 50). Baker’s cognitive model posited that early life stressors, such as childhood trauma, might lead to physical, psychological, and social dysfunction, increasing the risk of depression (51). According to that model, older adults with more CTEs would be more likely to experience stress and burdens, which adversely affects their daily lives and induces negative emotions, such as depression.

We also found that childhood trauma severity was associated with depressive symptoms in older adults, which extends others’ findings on the association between childhood trauma and depressive symptoms. The cognitive activation theory of stress (CATS) proposes that stress caused by external events requires an individual’s internal cognitive activation to have an effect (52). Based on the CATS theory, stress caused by CTEs might be determined by individual cognition, suggesting that studies on the association between CTEs and adverse health outcomes should evaluate the degree of the individuals’ perceived impact of CTEs, that is, childhood trauma severity. Older adults with more childhood trauma severity might experience sustained and long-term stimulation of CTEs, causing them to retain negative memories and experiences throughout their lives, which could induce negative emotions and depressive symptoms in old age. Researchers must consider assessments of childhood trauma severity to avoid overlooking their association with depressive symptoms. Therefore, our findings underscore that assessing only CTE occurrence or frequency might mask or reduce the association between childhood trauma and health outcomes. We suggest that the number of CTEs and childhood trauma severity should be evaluated together when studying childhood trauma to understand the potential impact of childhood trauma better. Meanwhile, considering both might provide a basis for developing more precise interventions.

We did not find that resilience could mediate the associations between CTEs and depressive symptoms, which aligns with a previous study (37), which reported that resilience did not mediate the association between ACEs and later-life depression. Although our results were similar, some differences in the assessment methods and concepts of resilience should be noted. Ward et al. conceptualized resilience as a response to stressful life events and used individuals’ feelings; they represented resilience using participants’ responses to seven stressful life events (37), which differed significantly from our study. We defined resilience as the ability to adapt and surmount adversity and traumatic events and used a standardized evaluation scale (CD-RISC-10), which systematic reviews have recognized as the highest-rated evaluation tool for resilience (34, 53). Nevertheless, we also found no significant mediating effect of resilience. Perhaps the effects of CTEs on geriatric depressive symptoms are buffered by social capital, such as social support and neighborhood cohesion (54), reducing the effect of resilience. Since only limited literature, further research exploring the mediating role of resilience in the association between childhood trauma event count and geriatric depression is required.

We also found that resilience mediated the association between childhood trauma severity and depressive symptoms in older adults, supporting the sensitization hypothesis of childhood trauma. This finding helps initially elucidate the mechanism underlying the association between childhood trauma severity and depressive symptoms. Stress process theory posits that stress might affect individual health through direct and indirect processes (by reducing positive psychological resources, such as resilience) (55). Based on that theory, older adults with higher childhood trauma severity might have poorer adaptation and coping ability to stress, causing them to develop negative emotions after experiencing stress and setbacks, thus resulting in depressive symptoms. Maercker et al. reported that higher childhood trauma exposure might predict resilience in older adults (56). Moreover, resilience is negatively associated with depression in older adults (57). These studies solidly support our findings.

In addition, we found no evidence that resilience moderates the associations of CTEs and childhood trauma severity with depressive symptoms, which is inconsistent with previous studies in Chinese adolescents (58). However, a US study also did not find a moderating effect of resilience between ACEs and depressive symptoms (59). In addition, a study in psychiatric nurses also found that resilience could mediate the relationship between occupational stress and mental health, but not moderate (60). The possible reason that the moderating effect of resilience was not found in the present study is that childhood trauma is a negative event early in life for older adults, who may be affected by these events throughout their life course, exposing older adults to prolonged stress. This long-term effect may extend beyond the protective effect of resilience on an individual’s mental health, rendering a moderating effect insignificant. A previous study in China found that resilience moderated the relationship between stressful life events that had occurred over the past year and depressive symptoms (61), which may be able to support our interpretation. As few studies currently focus on the role of resilience in the association between childhood trauma and adverse health outcomes in older adults, more studies are needed in the future to validate the findings of this study.

This study had the following advantages. First, we used standardized scales to assess CTEs and childhood trauma severity, enabling us to provide a more in-depth analysis of the association between childhood trauma and depressive symptoms in older adults. Second, this study was the first to identify the mediating and moderating roles of resilience in the association between childhood trauma severity and depressive symptoms. Our findings have significant implications for developing interventions to prevent depressive symptoms in older adults. Specifically, enhancing resilience might reduce the likelihood of older adults with severe childhood trauma developing depressive symptoms. A previous controlled clinical trial found that resilience training might significantly reduce individuals’ depressive symptoms and stress (62). A resilience intervention for young people with ACEs also showed improved health outcomes in the intervention group (63). However, few intervention studies have targeted older adults with CTEs. This study’s results underscore the need for additional intervention studies from a resilience perspective.

This study had some limitations. First, the cross-sectional study design could not infer a causal relationship between childhood trauma, resilience, and depressive symptoms in older adults. Second, the assessments of CTEs and childhood trauma severity were retrospective self-reports, which might be subject to selective recall, information biases, and may even result in the findings of the present study being biased. Therefore, more longitudinal studies are needed to validate the findings of the present study. In addition, Previous studies have shown that CTEs might also be associated with severe cognitive impairment and major depression (64, 65). We excluded individuals with severe cognitive impairment and major depressive disorder from this study because they could not complete the survey. This exclusion might have led to a selection bias. Third, all our participants were older adults from a single city in China. Future research should expand the sample area to determine whether our results can be generalizable to the entire Chinese older adult population.

## 5. Conclusion

CTEs and childhood trauma severity were all associated with depressive symptoms in older adults in China. Resilience mediated the relationship between childhood trauma severity and depressive symptoms. Intervention measures to improve resilience might help reduce the childhood trauma severity associated with depression risk for older Chinese adults.

## Data availability statement

The datasets that support the findings of this study can be made available to those interested by contacting the corresponding author.

## Ethics statement

The Medical Ethics Committee of the Second Affiliated Hospital of Shandong University of Traditional Chinese Medicine approved this study. The patients/participants provided their written informed consent to participate in this study.

## Author contributions

SL designed the study, completed the data analysis and manuscript writing. YtY and GC collected the data. CZ, HZ, and YY revised the manuscript. All authors contributed to the article and approved the submitted version.

## Funding

This study has received funding from National High Level Hospital Clinical Research (Number: BJ-2022-133).

## Conflict of interest

The authors declare that the research was conducted in the absence of any commercial or financial relationships that could be construed as a potential conflict of interest.

## Publisher’s note

All claims expressed in this article are solely those of the authors and do not necessarily represent those of their affiliated organizations, or those of the publisher, the editors and the reviewers. Any product that may be evaluated in this article, or claim that may be made by its manufacturer, is not guaranteed or endorsed by the publisher.
